# A case of cooperative breeding in the European Starling, *Sturnus vulgaris*

**DOI:** 10.1002/ece3.8318

**Published:** 2022-02-12

**Authors:** Hazel J. Nichols, Kevin Arbuckle

**Affiliations:** ^1^ Department of Biosciences Swansea University Swansea UK

**Keywords:** behavioral innovation, cooperative breeding, feeding rate, sturnidae

## Abstract

Cooperative breeding, where individuals other than the parents help to raise offspring, occurs in only ~9% of bird species. Although many starlings (Sturnidae) are cooperative breeders, the European starling (*Sturnus vulgaris*) has rarely been observed exhibiting this behavior. Only two other records exist, one of which was limited to a juvenile giving food to chicks that had already been collected by a parent (and hence providing limited help). Herein, we report a case of cooperative breeding by a juvenile European starling, which represents the second with any evidence of the juvenile collecting food independently and the first to document the extent of such help in the form of feeding rates. Over a period of at least 3 days, a juvenile starling assisted two parents to feed their second brood of the year, and it fed the chicks at the same rate as the adults (~3.5 feeds per hour). In considering potential explanations for this behavior, we conducted an ancestral state estimation of cooperative breeding across starlings and were able to eliminate the possibility that this is a rarely expressed behavior inherited from cooperatively breeding ancestors. Instead, we propose that our observations point to a behavioral innovation, which may be in response to environmental change such as climate change (which has previously been associated with cooperative breeding). Researchers working on birds should be alert to such behavior to determine whether this apparently new breeding strategy will increase as a potential adaptation to environmental change.

## Graphical abstract

Here, we report observations of cooperative breeding in European starlings—a rare reproductive strategy almost unknown in this common and well‐studied species. A juvenile from a first brood was observed over several days at a nest (presumably its parent's) actively and independently feeding offspring of a second brood at a rate comparable with the parents. This may represent the emergence of a novel behavioral trait, and hence, we encourage future monitoring to document the spread (if any) across populations of this species.

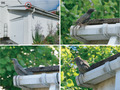

## 1 INTRODUCTION

Cooperative breeding, in which individuals other than the parents assist in the production of young, occurs in around 9% of birds (Cockburn, 2006). It often arises when offspring remain on their parents' territory after gaining nutritional independence and become “helpers at the nest,” assisting in rearing subsequent broods (Ligon & Burt, 2004). This helping behavior presents several potential costs, for instance, helpers sometimes forgo their own reproduction in order to help—usually gaining indirect fitness (if helping kin) but at a cost to direct fitness (Dickinson & Hatchwell, 2004). Importantly, even if no immediate opportunities for reproduction are lost, for instance, if the helper is too young to breed, helping can still have a negative impact on future reproduction. Feeding another brood is likely to be energetically costly due to nutritional resources being given to the brood rather than being consumed by the helper. This is probably why very young individuals rarely help (or help at low rates), even in species that commonly breed cooperatively (Heinsohn & Cockburn, 1994; Nichols et al., 2012). Hence, individuals helping when too young to breed are still potentially reducing the resources available to themselves to survive and develop into breeding adults and may therefore be losing future breeding potential.

As raising the offspring of others appears to be a Darwinian paradox, the evolution of cooperative breeding has received much attention and several benefits of becoming a helper have been identified. First, helpers may gain direct benefits from remaining on their natal territory. For example, philopatric offspring may receive less aggression from residents, have higher survival, and may inherit a breeding position if their parent dies or disperses (Ekman et al., 2004). Second, helpers may gain indirect fitness benefits from helping to rear kin, which are usually half or full siblings when helping takes place on the parents' territory (Dickinson & Hatchwell, 2004).

The factors that lead to the evolution of cooperative breeding are not fully understood. Both unpredictable environments (Jetz & Rubenstein, 2011) and predictable environments (Gonzalez et al., 2013) have been associated with this breeding strategy, dependent on the phylogeny and life history of the species concerned. The kin‐selected benefits of helping mean that genetic monogamy is important in the evolution of cooperative breeding, with helping behavior being more likely in species with low levels of promiscuity (Cornwallis et al., 2010; Lukas & Clutton‐Brock, 2012). Much variation also occurs between species in the frequency of cooperation; some being obligate cooperative breeders with pairs never breeding without helpers, while others show facultative cooperative breeding with some but not all pairs being assisted by helpers. Here, variation can occur within a population. For example, ~50% of long‐tailed tit nests have between one and five helpers (Hatchwell et al., 2004), whereas the remainder have no helpers. In other species, breeding strategies can vary between populations; for example, cooperative breeding is largely absent across most of the range of carrion crows, but some cooperative populations have been discovered (e.g., in Northern Spain; Baglione & Canestrari, 2016). To further understand the evolution of cooperative breeding and the factors that can promote it, it is important to record the distribution and frequency of helping within species.

Starlings provide an excellent taxonomic group to further our understanding of the evolution of cooperation. Cooperative breeding is relatively common in this group, especially in African starlings (Rubenstein & Lovette, 2007). European starlings *Sturnus vulgaris*, however, are not known to breed cooperatively, although they are well studied as they are common, widespread, and readily breed in nest boxes. They have 1–2 broods per year mostly between March and July, often with a single brood in the North of their range due to a colder climate and hence shorter warm season (Craig & Feare, 2009). European starlings nest in pairs (but nests may be clustered near each other) and they are predominantly monogamous, although a variety of other mating systems occur occasionally (Craig & Feare, 2009). Polygyny and extra‐pair paternity occur relatively frequently in some populations, with males occasionally having up to five mates (Pinxten et al., 1989), and clutches laid by one female may be fathered by up to three males (Pinxten et al., 1993). Intraspecific brood parasitism occurs at low frequency, with females laying eggs in the nest of conspecifics, sometimes removing a host egg at the same time (Lombardo et al., 1989). The combination of facultative extra‐pair paternity and intraspecific brood parasitism has led to cooperative nest defense by birds from neighboring nests, who may have some offspring in the nest under threat (Lewis, 2016). Communal breeding has been observed at least three times, where two females shared the same nest and both females and a single male (presumably the father) contributed to offspring care (Eens & Pinxten, 1993; Pinxten et al., 1994; Stouffer et al., 1988).

There are two previous observations of a nonbreeding helper at the nest in European starlings. In the Netherlands in 2015, a juvenile from a first brood was recorded helping its parents to feed a second brood on 3 days and, on at least one of these days, was gathering the food itself (the juvenile was also seen to beg for food from the parents and subsequently feed this to the nestlings in addition to eating some itself) (Ottens et al., 2016). The juvenile also took nesting material into the nest box and maintained the nest on 1 day. A report from the UK in 1975 also observed provisioning of a second brood of European starlings by an individual of the first brood, but in this case, the juvenile passed food given to it by the parents on to nestlings, rather than collecting the food itself (Warden, 1975). Here, we (1) report observations of a juvenile European starling feeding nestlings from a second brood for at least four consecutive days, (2) quantify feeding rates of the chicks by adults and the juvenile helper, and (3) determine whether the cooperative breeding we report may arise from cooperatively breeding ancestors in the lineage leading to European starlings.

## 2 METHODS

**FIGURE 1:**
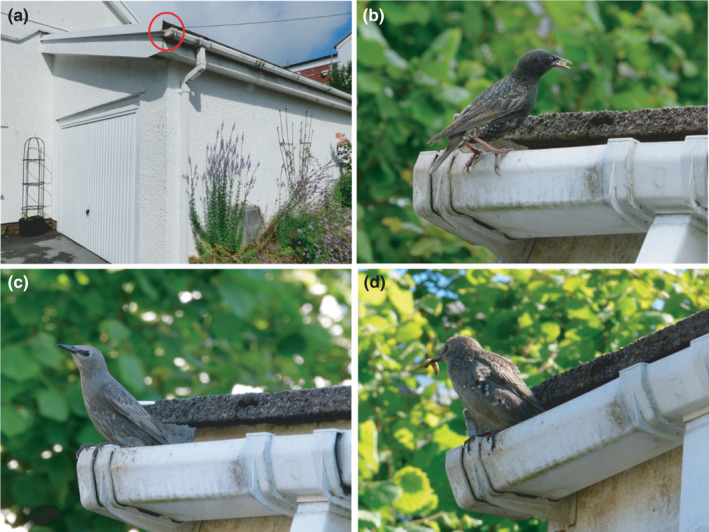
(a) The nest site, with the entrance located in the fascia to the garage (circled in red), (b) An adult at the nest entrance holding a piece of food, (c) The juvenile on 13th June, emerging from the nest after feeding the chicks, and (d) The same juvenile on 15th June (note the same pattern of postjuvenile molt of the breast feathers, but slightly further progressed) about to feed the chicks

We identified a European starling nest in a garage in a rural location on the Gower Peninsula, near Swansea, UK. The nest opened externally through guttering into the fascia approximately 2.5 m from the ground (Figure 1a). The nest had been loosely monitored from a distance throughout the spring, with begging calls from chicks and feeds from the adults being observed in April/May 2021 and the chicks fledging from the nest in May 2021.

The nest was subsequently re‐used, presumably by the same breeding pair, with chicks heard begging in early June 2021. On 12th June, we noticed a starling with juvenile plumage repeatedly visiting the nest, carrying food on entry but not on exit. Begging calls were heard from the chicks during each visit. We therefore have strong reason to believe that the juvenile, presumably a fledgling from the first brood at the same nest, was contributing to feeding the chicks of the second brood. We do not know how long the juvenile had been feeding the chicks prior to this date.

Formal observation of the nest began on 13th June, with observers sitting/standing approximately 7 m from the nest and recording the time of each feeding visit and whether the visit was by a juvenile or adult. We used a binomial test to investigate whether the rate of feeding by the juvenile was equivalent to that by the adults (assuming an equal share of feeding, 33%, by each bird). Observations were made for a mean of 122.4 min per day (range 46–293 min), predominantly in the afternoon/evening between 15.30 and 21.00, with the exception of 15th June which included a morning observation session from 10.30 to 12.00. Observations ended on 18th June, when the chicks fledged the nest. This study was approved by Swansea University College of Science Ethics Committee (SU‐Ethics‐Staff‐150621/370).

**FIGURE 2:**
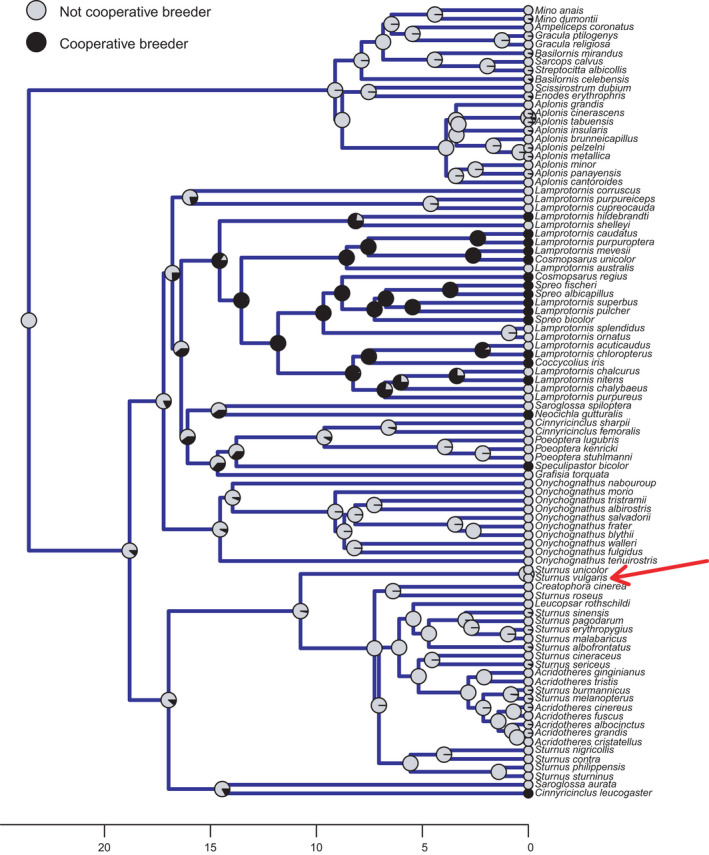
Phylogeny of starlings (Sturnidae) showing an ancestral state estimation of cooperative breeding. Pie charts at nodes (and tips) of the phylogeny show the probability of being a cooperative breeder (black) or not (light gray), and the x‐axis scale represents time in million of years before present. The red arrow indicates the European starling (*Sturnus vulgaris*)

To test whether a tendency for cooperative breeding in European starlings could be retained from behaviors of cooperatively breeding ancestors which are now rarely expressed, we also conducted an ancestral state estimation. Specifically, we first obtained 1000 phylogenies from the posterior distribution provided by birdtree.org (Jetz et al., 2012) for all members of the family Sturnidae with available genetic data. We then calculated the maximum clade credibility tree from this set using the phangorn v2.5.3 package (Schliep, 2011) in R v3.4.1 (R Core Team, 2017) and used that phylogeny for subsequent analysis. We collated data on whether or not each species is a cooperative breeder based initially on Rubenstein and Lovette (2007) for most African starlings and then supplemented with Craig and Feare (2009) for species not included in the former study. Where it was very unclear or there was no information, the data were treated as missing to ensure our analysis is robust to limited information for some species. Note that for this analysis, we did not consider the European starling as a cooperative breeder since this is not typical of its breeding strategy. This kept the data comparable with other species and therefore enabled us to better test the hypothesis that some ancestral behavioral tendencies may have been retained and expressed in the form of our observations. Ancestral states were estimated using Bayesian stochastic mapping (Bollback, 2006; Huelsenbeck et al., 2003) in the R package phytools v0.7.20 (Revell, 2012) based on 1000 simulations under an All Rates Different model with a root node prior estimated from the stationary distribution of the estimated transition matrix. Species with missing data were assigned an equal prior probability of being in each state (cooperative breeding or not) and their states were estimated within the analysis, all of which were resolved with high posterior probability (Figure 2).

## 3 RESULTS

The chicks were fed by one juvenile and two adults (Figure 1b–d). The adults were often difficult to differentiate from each other due to the brief nature of the feeding visits, so we did not distinguish between the adults in our records.

Between 13th and 15th June, the two adults and the juvenile attended the nest. During this time, the juvenile fed the nestlings as much as each adult did; on average, each adult gave 3.67 feeds per hour (total 51 feeds from adults), while the juvenile gave 3.31 feeds per hour (total 23 feeding visits). The juvenile therefore fed the offspring on 31% of all visits, which is not significantly different from the 33% expected if all three individuals shared feeding effort equally (binomial test, number of successes = 23, number of trials = 74, *p*‐value = .714). The juvenile did not usually arrive at the nest at the same time as adults and was not observed being given prey by the adults, strongly suggesting that the juvenile was collecting food by itself to feed the nestlings.

Three possible “false feeds” were observed. In one case, the juvenile landed on the garage roof carrying food but ate the food itself before entering the nest. In two cases, the adults returned to the nest without food or left with the food. There is therefore no evidence that the juvenile was more likely to false feed than the adults, although we cannot confirm whether or not individuals may have eaten the food themselves once they entered the nest.

The juvenile was last seen on 15th June at 20.25 on its final feeding visit of the day, and it was absent on 16th and 17th June despite 195 min of observation which included 58 feeding observations. It is unknown whether the juvenile died, dispersed, or simply stopped helping. The nestlings were observed to have fledged on 18th June.

We were able to confidently exclude one possible explanation for our results—that cooperative breeding is a (now) rarely expressed behavior inherited from cooperative ancestors. Our ancestral state estimation shows that European starlings are very unlikely to have any cooperative breeding in their evolutionary history (Figure 2), suggesting that a tendency for this behavior is not retained from cooperatively breeding ancestors.

## 4 DISCUSSION

We identify a case of cooperative breeding in European starlings, where a juvenile (presumed from the first brood) helped its parents to rear their second brood. This is only the third published case of a juvenile European starling helping its (presumed) parents to rear nestlings of a second brood, and the first paper to quantify feeding extent by the juvenile in comparison to the parents. Unlike the two previous observations of similar behavior (Ottens et al., 2016; Warden, 1975), the juvenile in our study appeared to gather the food itself, fed the brood consistently for at least 4 consecutive days (possibly for considerably longer), and fed at the same rate as the parents did. Hence, our observation appears to be distinct in that the juvenile was never observed being fed by the adults and was helping to rear the brood in a manner similar to other cooperative breeders.

It is currently unclear whether the cooperative breeding that we observed was a one‐off incident, or whether it occurs regularly in the local population. Helping behavior could potentially spread if the local environment favors it. For example, in carrion crows (*Corvus corone*), helping can be induced by cross‐fostering individuals from a noncooperative population in a cooperative population (Baglione et al., 2002). Genes are also likely to play a part in the evolution and maintenance of cooperative breeding, since a substantial heritable component to helping has been identified in western bluebirds *Sialia mexicana* (Charmantier et al., 2007) and Tibetan ground tits *Pseudopodoces humilis* (Wang & Lu, 2018), and lower but significant heritability exists in banded mongooses *Mungos mungo* (Nichols et al., 2021) and meerkats *Suricata suricatta* (Nielsen, 2013). Notably, we find no evidence that the observed cooperative behavior is a tendency inherited from a cooperatively breeding ancestor, and so if the behavior is more widespread than the observations reported here, then it is likely to be a recent behavioral innovation in this species.

Climate change may also play a part in the occurrence of helping behavior. As spring temperatures increase, European starling laying dates have been getting earlier (Both & te Marvelde, 2007). This may result in more pairs rearing second broods, thereby providing increased opportunities for help from the first brood. Moreover, although the mechanism remains unclear and perhaps variable between taxa, climate has been shown to be associated with cooperative breeding both within hornbills and across birds as a whole (Gonzalez et al., 2013; Jetz & Rubenstein, 2011). Considering that starlings are common and frequently observed birds and that the most similar observations to ours (Ottens et al., 2016) were as recent as 2015, it is possible that climate change is promoting the origin of novel behavioral strategies to cope with it, such as cooperative breeding, by selecting for such novelties once they arise. Alternative explanations which may favor cooperative behaviors include changes in habitat availability or predator communities, or effects of population size or density (via reduced habitat availability, changes to predation rates, etc.) which may lead to cooperative breeding as a result of limited availability of independent breeding opportunities (Komdeur, 1992). In our observations and the two previous reports, juveniles were helping to raise the brood, so breeding independently is unlikely to be constrained by increased competition in these cases. However, we do not have data that would allow us to robustly evaluate these various explanations. Future observations of the nest and wider population will reveal whether cooperation in European starlings represents a behavioral innovation that spreads further in the future.

The probability that helping behavior becomes more common in European starlings will likely be influenced by the fitness costs and benefits of providing help in the current and future environments. The juvenile in our observations was probably helping to rear full siblings so it may have gained indirect fitness as a consequence and also could have gained direct fitness in the form of experience that may enhance future breeding attempts. As the juvenile was too young to breed, it did not lose current breeding opportunities through cooperating, as may occur in species where sexually mature individuals help (Dickinson & Hatchwell, 2004). However, helping may have resulted in other costs; for example, feeding chicks is likely to be energetically expensive and can result in reduced growth (Heinsohn & Legge, 1999). It is not clear why the juvenile suddenly stopped helping, but it is possible that this was due to mortality, the probability of which could have been influenced by helping.

Despite being a well‐known species, our observations represent only the third observation of nestling feeding by a juvenile European starling, the second with any evidence of the juvenile collecting food independently, and the first to document the extent of help (feeding rate) and with no suggestion of merely passing on food given by parents. We believe this represents a case of cooperative breeding in this species which appears to be a behavioral innovation. Further observations of European starling nests containing a second brood will help to confirm whether this is a rare and ephemeral example or whether cooperative breeding will spread as a novel behavior in the wider population.

## CONFLICT OF INTEREST

We have no conflicts of interest to declare.

## AUTHOR CONTRIBUTIONS

**Hazel J. Nichols:** Conceptualization (equal); data curation (equal); formal analysis (equal); methodology (equal); visualization (equal); writing–original draft (lead); writing–review and editing (supporting). **Kevin Arbuckle:** Conceptualization (equal); data curation (equal); formal analysis (equal); methodology (equal); visualization (equal); writing–review and editing (lead).

## Data Availability

Data on observations and for the ancestral state estimation are available at https://doi.org/10.6084/m9.figshare.14987175.v1.
